# Platelet-rich fibrin vs. buccal advancement flap for closure of oroantral communications: a prospective clinical study

**DOI:** 10.1007/s00784-022-04846-7

**Published:** 2023-01-06

**Authors:** Stefan Hunger, Stefan Krennmair, Gerald Krennmair, Sven Otto, Lukas Postl, Danilo-Marc Nadalini

**Affiliations:** 1 Medical Faculty, Johannes Kepler University Linz, Altenberger Strasse 69, 4040 Linz, Austria; 2 Clinic of Oral and Maxillofacial Surgery, Kepler University Hospital, Johannes Kepler University, Krankenhausstraße 7a, Linz, Austria; 3 NumBioLab, Ludwig-Maximilians University of Munich, Munich, Germany; 4 Head of Department of Prosthodontics, Sigmund Freud University Vienna, Freudplatz 1, 1020 Vienna, Austria; 5 Department of Oral and Maxillofacial Surgery, Ludwig-Maximilians-University Munich, Lindwurmstr. 2a, 80337 Munich, Germany

**Keywords:** Platelet-rich fibrin, Buccal advancement, Closure of oroantral communications, Healing, Mucogingival border, Lukas Postl and Danilo-Marc Nadalini contributed equally to this work.

## Abstract

**Objectives:**

The primary aim was to evaluate the success of the defect closure (tight or open) of oroantral communications (OAC) after treatment with platelet-rich fibrin (PRF) clots or a buccal advancement flap (BAF). Secondary outcome measurements were the evaluation of the wound healing, the displacement of the mucogingival border (MGB), and the pain level.

**Material and methods:**

Fifty eligible patients with an OAC defect larger than 3 mm were randomly assigned to either PRF (test group, *n* = 25) or BAF (control group, *n* = 25) for defect closure. In a prospective follow-up program of 21 days, the defect closure healing process, the wound healing course using Landry’s wound healing index (score: 0–5), the displacement of the MGB, and the postoperative pain score were evaluated.

**Results:**

Five patients in each group were lost to follow-up resulting in 40 patients (20 in each group) for continuous evaluation. On postoperative day 21 (study endpoint), no difference regarding success rate (defined as closure of OAC) was noticed between the test (90%; 18/20) and control group (90%; 18/20). A univariate analysis showed significant differences for age and defect size/height for the use of PRF between successful-tight and open–failed defect healing. At the final evaluation, a significantly (*p* = 0.005) better wound healing score, a lower displacement of the MGB as well as lower pain-score were seen for the use of PRF.

**Conclusions:**

Based on the findings of the current study, the use of platelet-rich fibrin represents a reliable and successful method for closure of oroantral communications. The use of PRF clots for defect filling is associated with lowered pain levels and less displacement of the mucogingival border.

**Clinical relevance:**

The defect size should be taken into account when choosing the number and size of PRF plugs.

## Introduction

Oroantral communications (OAC) are unnatural openings between the oral cavity and the maxillary sinus, which occur due to loss of the soft and/or hard tissue separating these compartments [1]. The most common causes for OAC are tooth extractions in the posterior area of the maxilla, where there is a close topographical relationship between the root apices and the maxillary antrum. OAC may also form following the removal of maxillary cysts, tumors, facial traumata or during dentoalveolar and implant surgery [2–4]. When closure of an OAC fails, the opening may become epithelialized and develop into an oroantral fistula (OAF). This persistent communication between the oral cavity and the maxillary sinus can act as a pathway for bacterial and fungal penetration and has been frequently reported to induce maxillary sinusitis [5, 6].

Numerous treatment strategies have been developed for the management of OAC and OAF and have shown high rates of successful defect closure [7]. To date, the most commonly used method is a surgical approach using a buccal advancement flap (BAF), a technique which was first introduced by Rehrman in 1936 and has become the method of first choice for many clinicians, mainly due to its simplicity, reliability, and versatility [8–11]. Further popular types of local flaps are buccal fat-pad flaps and palatal flaps, but also free mucosal grafts, distant flaps such as tongue flaps, and the use auricular cartilage or bone grafts (e.g., from the chin or retromolar region) have proven to be viable methods for OAC/OAF closure [4]. However, postoperative pain and swelling are potential complications following all surgical options, but a specific drawback of local buccal flaps such as the BAF is vestibular shortening and loss of keratinized gingiva, which may have an impact on future prosthetic rehabilitation [7].

Recently, considerations of alternative treatment methods have included the successful use of plasma-rich fibrin (PRF) for OAC as reported by Demetoglu et al. [12], Assad et al.[13] and Bilginalyar [14]. The clinical outcome of OAC closure using PRF and BAF was compared in a randomized evaluation by Bilginaylar [15], and both methods were proven to be successful. However, postoperative pain and swelling was reduced when PRF was used. Furthermore, there is evidence that PRF has a beneficial effect on wound healing, which makes this method attractive for OAC/OAF closure. Previous studies have shown that wound healing after tooth extraction improved when the extraction socket was filled with PRF [16–19]. Although most case-series and comparative studies have evaluated the results of OAC closure by using clinical assessment only, there is still a lack of information about the detailed course of wound healing, for example by using a defined healing index. Based on several clinical parameters (redness, presence of bleeding, granulation tissue, epithelialization, suppuration), the wound healing index of Landry et al. [20] provides ratings from very poor to excellent healing and allows the evaluation of the wound healing process for comparing the effect of different treatment methods [17–19]. This index has been previously used in the report by Srinivas et al. [21] for judging the course of the wound healing after treating post-extraction sockets with PRF. Further parameters, which have not yet been evaluated and compared in detail for the use of PRF vs. BAF for OAC closure, are the displacement of the mucogingival border as well as the subjective patient-rated perception of pain and swelling.

As there is a lack of clinical data on OAC defect closure after PRF treatment, this study was conducted and focuses on two major aims: 1) The primary aim is to compare the wound healing outcome of the OAC defect closure with clinical parameters after treatment with PRF clots or after surgical treatment with a BAF. 2) The secondary aim is to assess the patient-related and surgery-related risk factors affecting the healing process and to determine the wound healing course by using a specific wound healing index. In addition, the displacement of the mucogingival line and the patient-scored pain level for both groups are evaluated. The initial working hypothesis is that the use of PRF and the buccal flap will not differ for the final clinical wound healing outcome of OAC defect coverage, but may be different regarding the wound healing course, the displacement of the mucogingival borderline and patient-related postoperative perception.

## Material and methods

### Study design—patient selection

The study was designed as a single-center, prospective, randomized, controlled follow-up study and was conducted at the author’s institution. During the period between December 2019 and July 2020, 50 consecutive patients (Table 1) were included for surgical OAC closure. Depending on the used defect coverage method, the patients were randomly assigned to either the PRF group (test group, *n* = 25) or to the BAF group (control group; *n* = 25). Randomization for the treatment method was performed using a permuted block-randomization approach (block length either 2, 4, or 6) without stratification and allocation to groups was done preoperatively by the sequentially numbered opaque sealed envelope (SNOSE) technique. For creating the randomization list, the R package “blockrand” (blockrand: Randomization for Block Random Clinical Trials, Greg Snow, R package version 1.5) was used. Each patient was given a detailed description of the procedure and signed an informed consent document prior to inclusion in the follow-up program. The study protocol had been approved by the local ethics committee (EC No: 1192/2019) and the study was conducted in accordance with good clinical practices and the Declaration of Helsinki. The study was self-funded by the authors and their institution.

**Table 1:** Exclusion criteria

Patient-specific	Diabetes with HbA1c > 7,5%
	Use of antirheumatic drugs, antiresorptive medications (such as bisphosphonates) or corticoids
Anatomical-specific and surgery-specific	Oroantral communication due to other reasons (traumatic, neoplastic,…)
	Communications with a diameter < 3 mm
	Recurrence of a communication (reoperation is necessary)

### OAC: diagnosis–morphology–location

The diagnosis for OAC was initially assessed by clinical examination using a conventional blunt probe (diameter: 1 mm) and by the patient performing the Valsalva pressure maneuver. The blunt probe was used to carefully palpate the defect in order to detect an OAC. The Valsalva pressure maneuver was additionally performed on each patient and in case of air leakage from the maxillary sinus, an OAC was also assumed. In case of a palpable defect, an apical perforation size of at least 3 mm was assessed using a 3 mm probe. In the further course, the perforation size was verified using detailed radiographic evaluation such as CBCT. The defect area, the mesial and the distal defect length were calculated. The morphology of the OAC was defined in a parallel-walled, root-shaped and inverse-root-shaped (higher apical width) design. Randomization for the treatment method was performed using a permuted block-randomization approach (block length either 2, 4, or 6) without stratification. For creating the randomization list, the R package “blockrand” (blockrand: Randomization for Block Random Clinical Trials, Greg Snow, R package version 1.5) was used.

### Surgical approach

According to the randomization protocol, surgical closure of OAC was performed using either platelet-rich fibrin (PRF; test group, *n* = 25) or the buccal advancement flap (BAF; control group, *n* = 25). All procedures were performed by the same experienced surgeon (S.H.) under local anesthesia (Xylanest® 2%; Gebro Pharma). Postoperatively, patients in both groups were given amoxicillin (Amoxicillin/Clavulanic Acid 875 mg/125 mg or Clindamycin 600 mg) three times/day for 7 days and ibuprofen 600 mg (if needed) and were instructed to rinse their mouth (2 weeks/0.12% chlorhexidine gluconate solution/twice daily) and to use a nasal decongestant (Fentrinol®, twice daily for 5 days). They were also advised not to blow their noses for 21 days.

### Platelet-rich fibrin (PRF)—test group

**Fig. 1:**
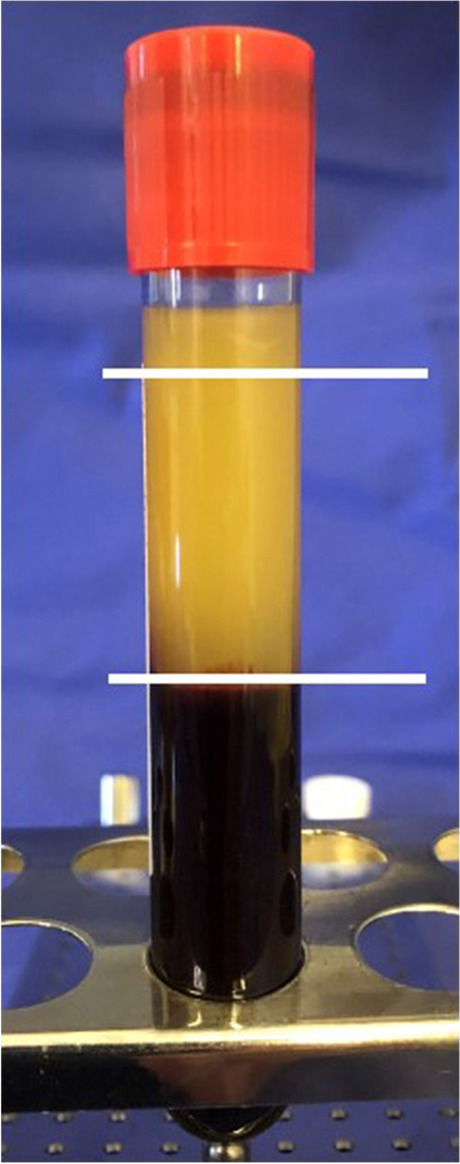
Picture during treatment shows the production of PRF: Centrifugation results in a fibrin clot (PRF) in the center of the tube. Red blood cells collect at the base of the tube and acellular plasma is located at the top layer of the tube (The three areas are indicated by white lines)

**Fig. 2:**
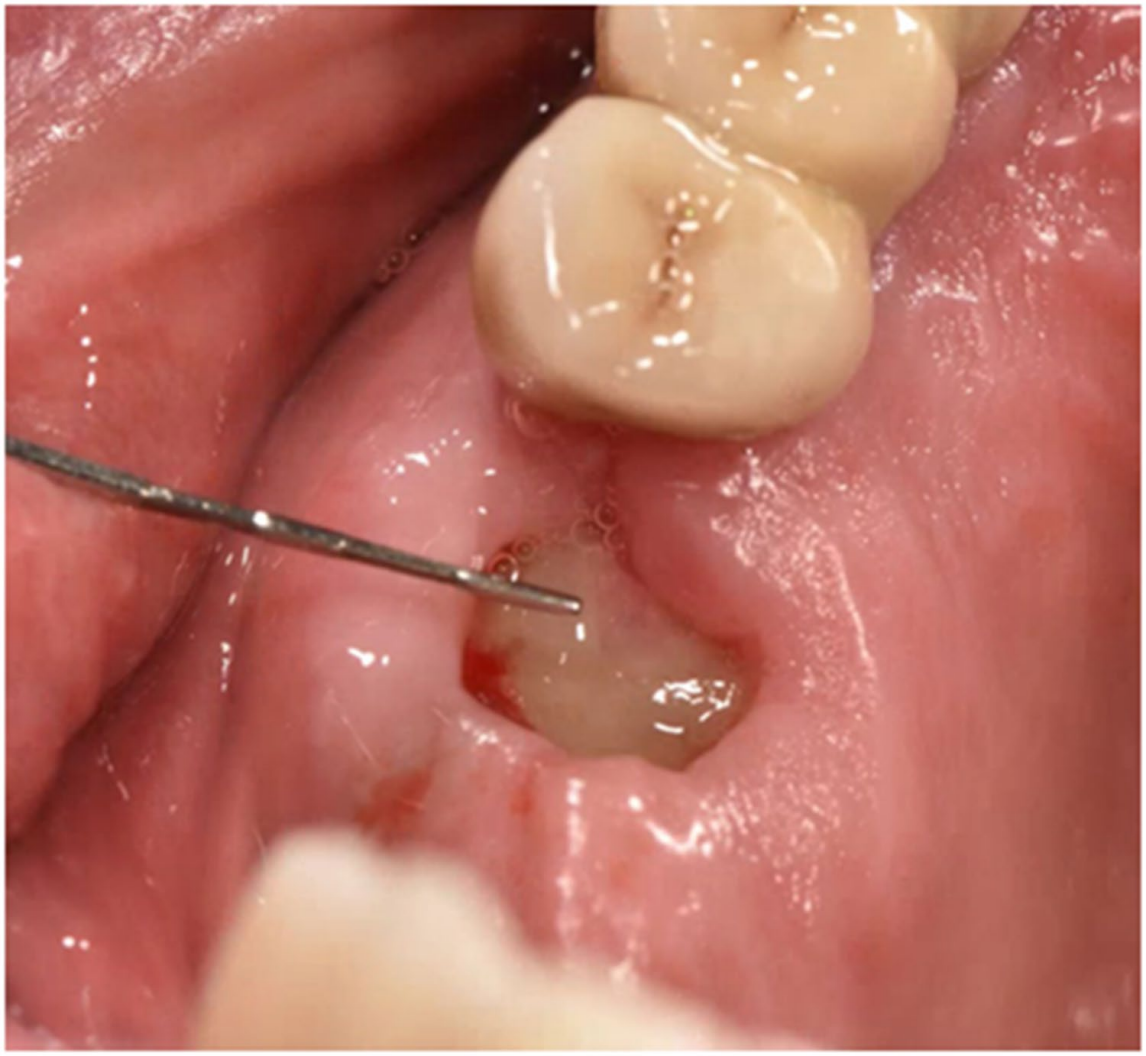
Clinical picture during treatment: an extraction socket in region 26 is shown directly after the placement of a PRF clot

According to the protocol of Choukron’s [22] PRF production, 4 glass tubes of 10 ml of patient venous blood were centrifuged (1300 revolutions/8 min at 210 G) for clot production. Figure 1 shows a glass tube after centrifugation. Two PRF clots were condensed by stamp pressure and two PRF clots were formed into membranes. PRF clots were inserted into the OAC defect (Fig. 2) and fixed on mucosal margins with absorbable sutures (Vicryl® 4–0) preventing dislocation in the maxillary sinus. Both PRF membranes were placed in two layers over the PRF clots and were also fixed to the marginal gingiva with a Laurell suture (absorbable, Vicryl 4–0).

### Buccal advancement flap (BAF)—control group

A trapezoid full mucoperiosteal flap was prepared [8–11] consisting of a crestal incision with a mesial and distal release suitable for flap elevation. For tension-free wound closure, a basal periosteal releasing incision parallel to the coronal margin was performed. Before wound closure an incision removal of the epithelial lining of the oroantral communication was done. The trapezoidal flap, consisting of epithelium, connective tissue and periosteum, was positioned over the defect by means of holding stitches (Vicryl 4/0 DA 0.45 m, Johnson & Johnson, New Brunswick, New Jersey, USA) from the buccal flap to the palatal mucosa. Final adaptation was made with single sutures (Vicryl 4/0 DA 0.45 m, Johnson & Johnson, New Brunswick, New Jersey, USA).

### Clinical analyses

The patients were regularly and prospectively followed up with recalls on the postoperative days 7 and 21. Additional recall visits were scheduled, if any clinical problems were encountered. All follow-up visits comprised evaluation of the clinical healing outcome such as defect tightness (primary outcome measurement) for the defect closure of oroantral communication using PRF (test group) or buccal advancement flap (BAF; control group). Secondary outcome measurements included -1) evaluation and comparison of patient-related, surgery-related or anatomical risk factors affecting the wound healing process for OAC treated with PRF or BAF, -2) comparison of the wound healing course between PRF and BAF using a scoring system with defined healing indices, -3) evaluation of the displacement of mucogingival line as well as -4) evaluation of postoperative pain and of the use of painkillers.

### Primary outcome measurement

**Fig. 3:**
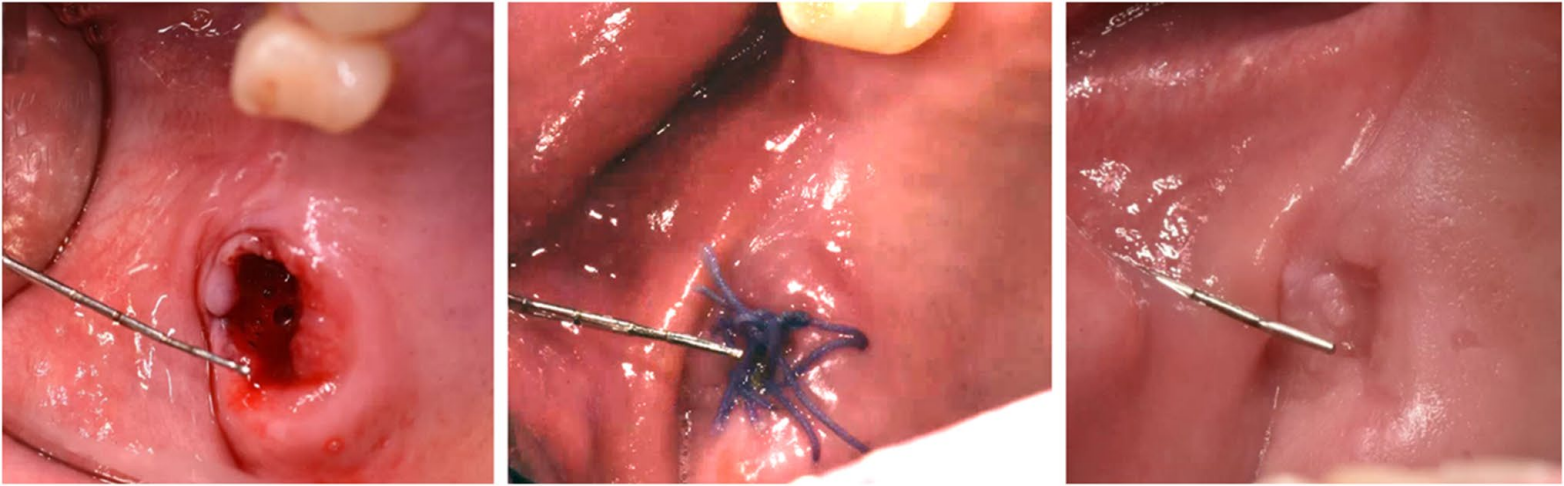
Patient of the PRF group with an OAC in region 27 (left side: postoperatively, center: 1 week post op, right side: 3 weeks post op)

**Fig. 4:**
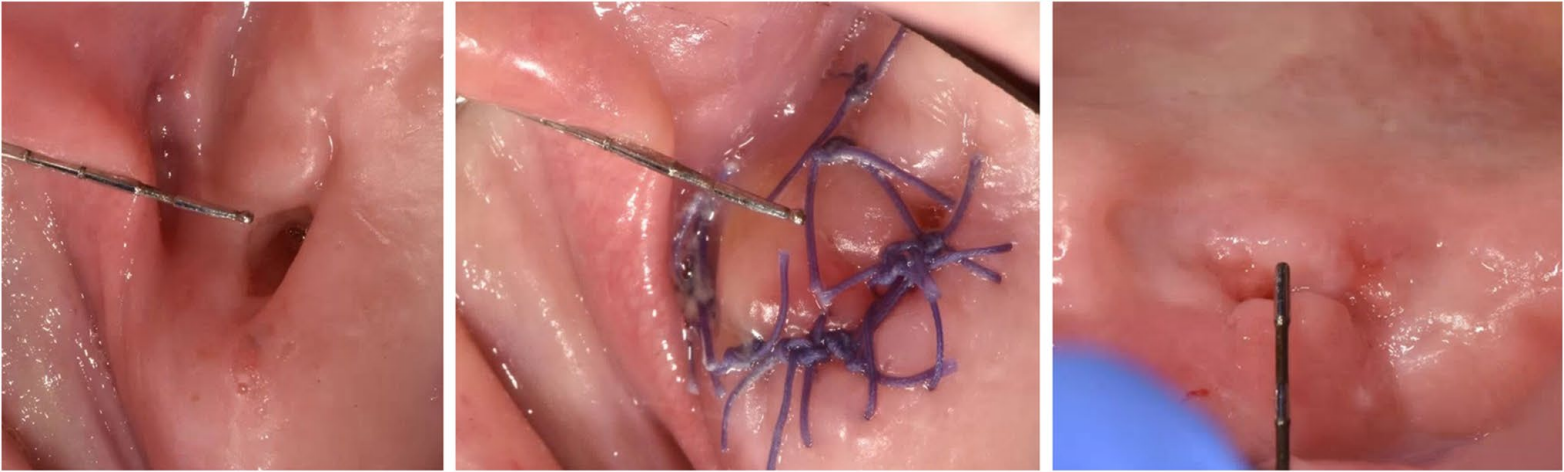
Patient of the BAF group with an OAC in region 16 (left side: postoperatively, center: 1 week post op, right side: 3 weeks post op)

The assessment of the primary outcome measurements during the first 21 days included the evaluation of the clinical outcome (tight or open) of the defect closure process for both groups. Figure 3 shows an example from the PFR group, Fig. 4 an example from the BAF group. The tightness of the defect closure was checked by applying a plunger probe (1 mm diameter at the apex) with gentle pressure against the newly formed tissue on postoperative day 7 and day 21. Tightness of the defect closure was also assessed using a dichotomous score (yes = 1; no = 0) and was compared between both groups.

### Secondary outcome measurements

1. Patient-related and surgical-anatomical risk factors were evaluated and compared for the presence of successful (tight) defect closure or open defect (failed-healed) OAC for the study endpoint (evaluation at day 21).
2. Clinical wound healing: Using the wound healing index of Landry et al., the course of wound healing of OAC closure was assessed on day 7 and day 21 in the PRF and BAF group. Two independent assessors (M.M.; G.F.) not involved in the surgical procedure rated the following five items dichotomously (0/1) resulting in a summarized score ranging from 0 (worst) to 5 (highest).
3. color of the surrounding mucosa: inflammatory red (score: 0) / healthy pink (score: 1).
4. bleeding on palpation: presence = 0 / absence = 1
5. granulation tissue: presence = 0 / absence = 1
6. incision margin/re-epithelialization: presence = 0 / absence = 1
7. suppuration: presence = 0 / absence = 1

-3) Offset of the mucogingival line: The mucogingival borderline was initially measured from the jaw ridge center to the mucogingival borderline in millimeters using a periodontal probe. Measurements of the distance were done preoperatively (baseline) and on postoperative days 7 and 21 and were compared within the groups as well as between the PRF and BAF group.

-4) Postoperative pain/use of painkillers. All patients received a pain protocol for assessing pain level and the pain medications taken daily for the first seven days. Pain level was assessed with a visual analog scale ranging from 0 (= no pain) to 10 (= severe pain). Pain level was followed up for 7 days and was compared between the test group and the control group. In addition, the number of painkillers used was also assessed for 7 days and evaluated for both groups.

### Statistical analysis

All statistical tests and confidence intervals were used in an explorative way, therefore no correction of the type I error (two-sided, 5%) was made except for post-hoc comparisons. All results are therefore descriptive. For all statistical analyses, the open-source statistical computing software R Version 4.1.2 (R Foundation for Statistical Computing, Vienna, Austria. URL http://www.R-project.org) was used.

Fisher’s exact test (2 × 2 tables) or the exact Chi-Square test (n x k tables) were used for unpaired categorical variables. In the case of two independent groups of ordinal variables the exact Mann–Whitney *U* test was used. As normal-distribution-test for continuous variables, the Kolmogorov–Smirnov-Test with Lilliefors Correction at a type-I error-rate of 10% was used. As test of variance homogeneity for continuous variables, the Levene-Test was used at a type-I error-rate of 5%. In the case of two independent groups the unpaired two sample t-test respectively Welch’s two sample *t*-test in case of variance heterogeneity was used. In case of not normally distributed data the exact Mann–Whitney *U* test was used. For paired normally distributed data (verification with Kolmogorov–Smirnov-Test with Lilliefors Correction at a type-I error-rate of 10%) the paired t-test was used. If the paired data was not normally distributed the exact Wilcoxon test was used. In case of more than 2 repeated measurements repeated ANOVA (post hoc comparisons using Bonferroni-adjusted paired t-tests) for normally distributed data or Friedman’s rank analysis of variance (post hoc comparisons using the Schaich-Hamerle approach) for not normally distributed data was used.

For assessing the equivalence regarding the outcome “defect closure after 21 days,” an exact two-sided 90% confidence interval for the difference of the proportion of defect closure with an equivalence region of ± 20% was used. The relationship between a metric variable and dichotomous variable was estimated by the point biserial Bravais-Pearson-correlation-coefficient or by the point biserial Spearman-correlation-coefficient (in case of no normal distribution). As test for the correlation coefficient against the reference value zero (no correlation), a test based on the t-distribution was used.

## Results

### Patients

Out of the 50 patients originally included (*n* = 50), five patients in the test group (5/25; 20%) and five patients in the control group (5/25; 20%) were lost to follow-up due to lack of compliance with continuous evaluation. In detail, 3 patients were lost on day 7 (3 × control group; BAF) and 7 patients (5 in test group [PRF], 2 in control group [BAF]) were lost on day 21 resulting in 10 patients (5 in each group) without continuous follow-up. The reasons for the drop-out varied and primarily involved the extramural follow-up setting. Finally, 20 patients in the test group and 20 patients in the control group could be continually followed on the postoperative days 7 and 21 (final endpoint evaluation) and thus contributed to the evaluation of primary and secondary outcome measurements. Table 2 shows the patient characteristics such as age, gender, body mass index along with the *p*-values indicating that there were no significant differences between the test and control group for each parameter. Defect area and defect morphology of test and control group are shown in Table 3.

**Table 2:** Patient characteristics of all patients. Data are given as mean ± standard deviation or absolute/relative frequencies

	PRF (*n* = 25)	BAF (*n* = 25)	*p*-value
Patient-related			
age	47.0 ± 14.9	51.6 ± 10.9	0.226
sex (f/m)	11(44%)/14(56%)	12(48%)/13(52%)	> 0.999
BMI	26.9 ± 4.9	24.7 ± 4.6	0.066
Smoking	7(28%)	9(36%)	0.762

**Table 3:** Defect area and morphology. Data are given as mean ± standard deviation or absolute/relative frequencies

	PRF (*n* = 20)	BAF (*n* = 20)	*p*-value
Anatomical site related			
localisation first premolar	0(0%)	0(0%)	> 0.999
localisation second premolar	1(5%)	1(5%)	> 0.999
localisation first molar	10(50%)	11(55%)	> 0.999
localisation second molar	6(30%)	3(15%)	0.451
localisation wisdom tooth	3(15%)	5(25%)	0.695
defect size in mm^2^	40.5 ± 33.1	54.6 ± 32.6	0.108
mesial bone height in mm	7.5 ± 2.4	5.0 ± 3.3	0.008
distal bone height in mm	6.4 ± 2.4	4.8 ± 2.9	0.065

### Primary outcome—defect closure

**Fig. 5:**
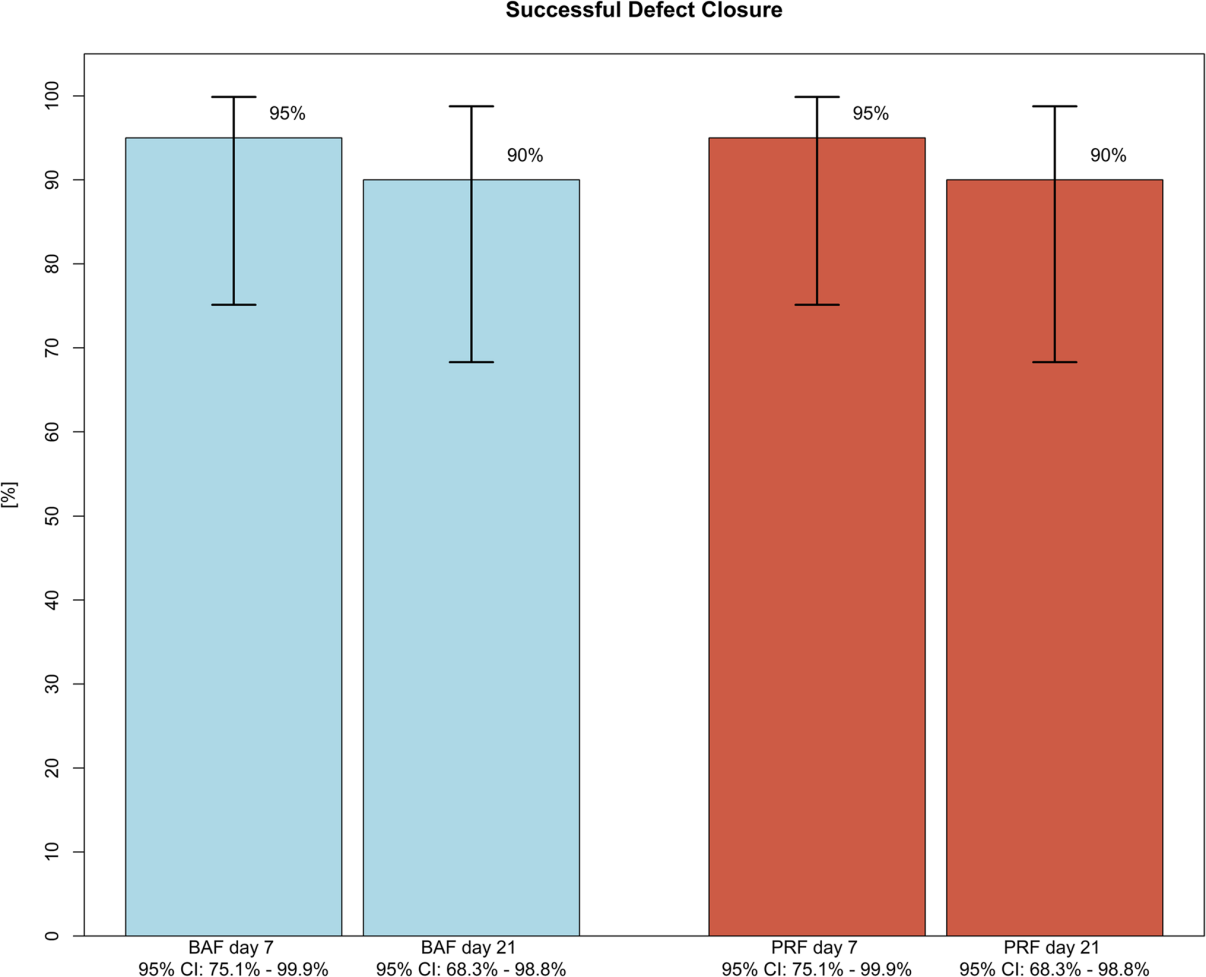
Success of defect closure

The day-7 follow-up evaluation of the defect closure of the oroantral communication showed a success rate of 95% (19/20) for the PRF group and 95% (19/20) for the BAF group. Evaluation on postoperative day 21 (study endpoint) revealed a success rate of 90% (18/ 20) for both the test and the control group. Figure 3 shows an example from the PFR group, Fig. 4 an example from the BAF group. Figure 5 presents information about successful defect closure including exact 95% confidence intervals. Considering the hypothesized absence of any difference between the two groups regarding successful defect closure, a statistically relevant equivalence of both groups (*p* = 0.043; 90% CI: − 19.69– + 19.69%) based on an equivalence region of 20% was found.

### Secondary outcomes

#### Patient-related and surgical risk factors

Table 4 shows the univariate risk factor analysis for patient-related and surgical/anatomical risk factors for the use of PRF or BAF with tight-successful or open-failed conditions of OAC closure at the time of the endpoint evaluation (day 21). There were significant differences for age, defect size/height for the use of PRF between successful-tight and open–failed defect healing. In addition, a moderate positive correlation (*r* = 0.491) between defect size and occurrence of a persistent oroantral connection was found in the PRF group after 21 days (*p* = 0.028). In contrast, at the endpoint evaluation (at day 21) no significant differences between successful and failed healing outcome were found for the risk factors when BAF was used.

**Table 4:** Univariate risk factor analysis for patient-related and surgery-related/anatomical risk factors for using PRF or BAF. Data are given as mean ± standard deviation or absolute/relative frequencies

		PRF day 21			BAF day 21	
	OAC-closed	OAC –open	p-value	OAC-closed	OAC –open	*p*-value
	*n* = 18	*n* = 2		*n* = 18	*n* = 2	
Sex			0.479			0.189
f	9 (50.0%)	0 (0.0%)		7 (38.9%)	2 (100%)	
m	9 (50.0%)	2 (100%)		11 (61.1%)	0 (0.0%)	
Age	46.2 ± 15.1	68.0 ± 5.7	0.037	50.8 ± 10.6	60.5 ± 6.4	0.258
BMI	26.91 ± 5.3	30.1 ± 5.4	0.442	25.4 ± 5.1	20.5 ± 1.1	0.095
Area	32.41 ± 16.8	113.1 ± 66.2	0.021	51.7 ± 33.1	80.4 ± 1.0	0.379
mesial bone height	8.0 ± 1.9	2.9 ± 0.04	0.011	5.2 ± 3.3	3.4 ± 3.2	0.442
distal bone height	6.8 ± 2.1	3.1 ± 2.9	0.126	4.8 ± 2.9	4.8 ± 4.9	0.937
Side			0.495			> 0.999
right	10 (55.6%)	2 (100%)		11 (61.1%)	1(50.0%)	
left	8 (44.4%)	0 (0.0%)		7 (38.9%)	1(50.0%)	
Location						
First premolars	0(0.0%)	0(0.0%)	> 0.999	0 (0.0%)	0 (0.0%)	> 0.999
Second premolars	1(5.6%)	0(0.0%)	> 0.999	1 (5.6%)	0 (0.0%)	> 0.999
First molars	9(50.0%)	1(50.0%)	> 0.999	9 (50.0%)	2 (100%)	0.479
Second molars	5(27.8%)	1(50.0%)	0.521	3 (16.7%)	0 (0.0%)	> 0.999
Wisdom teeth	3(16.7%)	0(0.0%)	> 0.999	5 (27.8%)	0 (0.0%)	> 0.999
Morphology			0.132			0.479
Parallel	5 (27.8%)	0 (0.0%)		4 (22.2%)	0 (0.0%)	
Root-shaped	9 (50.0%)	0 (0.0%)		5 (27.8%)	0 (0.0%)	
Inverse-root	4 (22.2%)	2 (100%)		9 (50.0%)	2 (100%)	
Smoker			> 0.999			> 0.999
No	13 (72.2%)	1 (50.0%)		12 (66.7%)	1 (50.0%)	
Yes	5 (27.8%)	1 (50.0%)		6 (33.3%)	1 (50.0%)	
Closing attempts			0.016			0.195
First closing attempt	17 (94.4%)	0 (0.0%)		17 (94.4%)	1 (50.0%)	
Revision	1 (5.6%)	2 (100%)		1 (5.6%)	1 (50.0%)	

#### Wound healing according Landry index

**Fig. 6:**
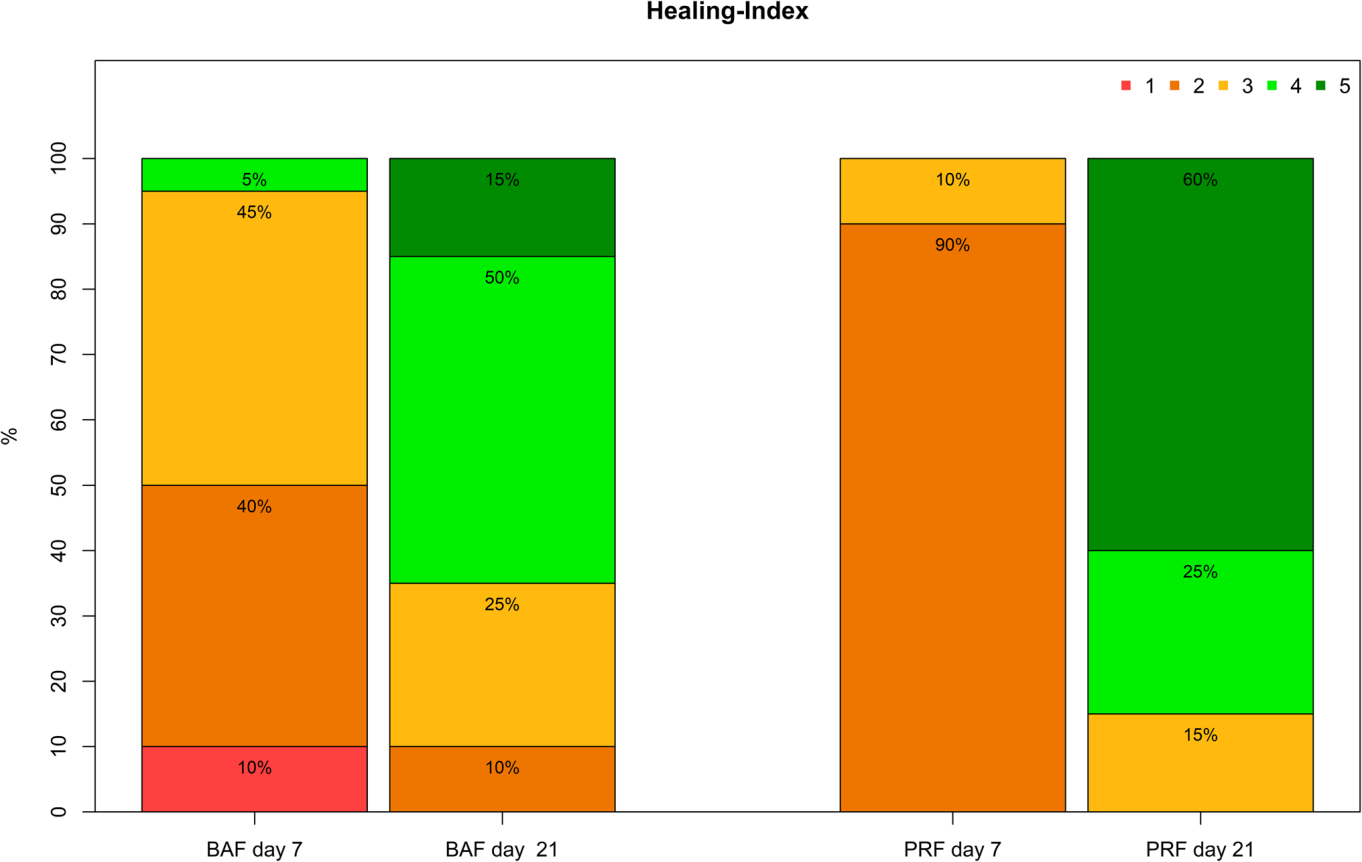
Clinical healing course for the test and the control group on day 7 and day 21 evaluated using the Landry wound healing index

The results for the clinical healing course in the test and the control group on day 7 and day 21, which were evaluated using the Landry wound healing index, are shown in Fig. 6.

For both groups (PRF and BAF) a significant increase (*p* < 0.001) of healing was noticed between day 7 and day 21. Initially, at day 7 a better healing score was observed for the use of BAF (2.5 ± 0.8) than for PRF (2.1 ± 0.3). However, on day 21 an inverse healing course was seen showing a significantly (p = 0.005) better wound healing score for the use of PRF (4.5 ± 0.8) than for the use of BAF (3.7 ± 0.9).

#### Displacement of MGL

Clinical displacement of the mucogingival line in the PRF and the BAF group at baseline (preoperatively), on day 7 and day 21 is shown in Table 5. Regarding the values related to the mucogingival line, there was a significant overall difference (*p* < 0.001) in the BAF group, which was caused by significantly lower values on day 7 (*p* < 0.001) and day 21 (*p* < 0.001) compared to baseline. For the PRF group no post-hoc significances were observed.

**Table 5:** Clinical displacement of the mucogingival line (MGL). Data are given as mean ± standard deviation

	PRF (n = 20)	BAF (n = 20)	*p*-value
MGL: preoperatively	9.1 ± 2.4	8.3 ± 2.4	0.301
MGL: at day 7	9.1 ± 2.4	2.1 ± 2.3	< 0.001
MGL: at day 21	8.9 ± 2.3	1.8 ± 2.1	< 0.001

#### Postoperative discomfort

**Fig. 7:**
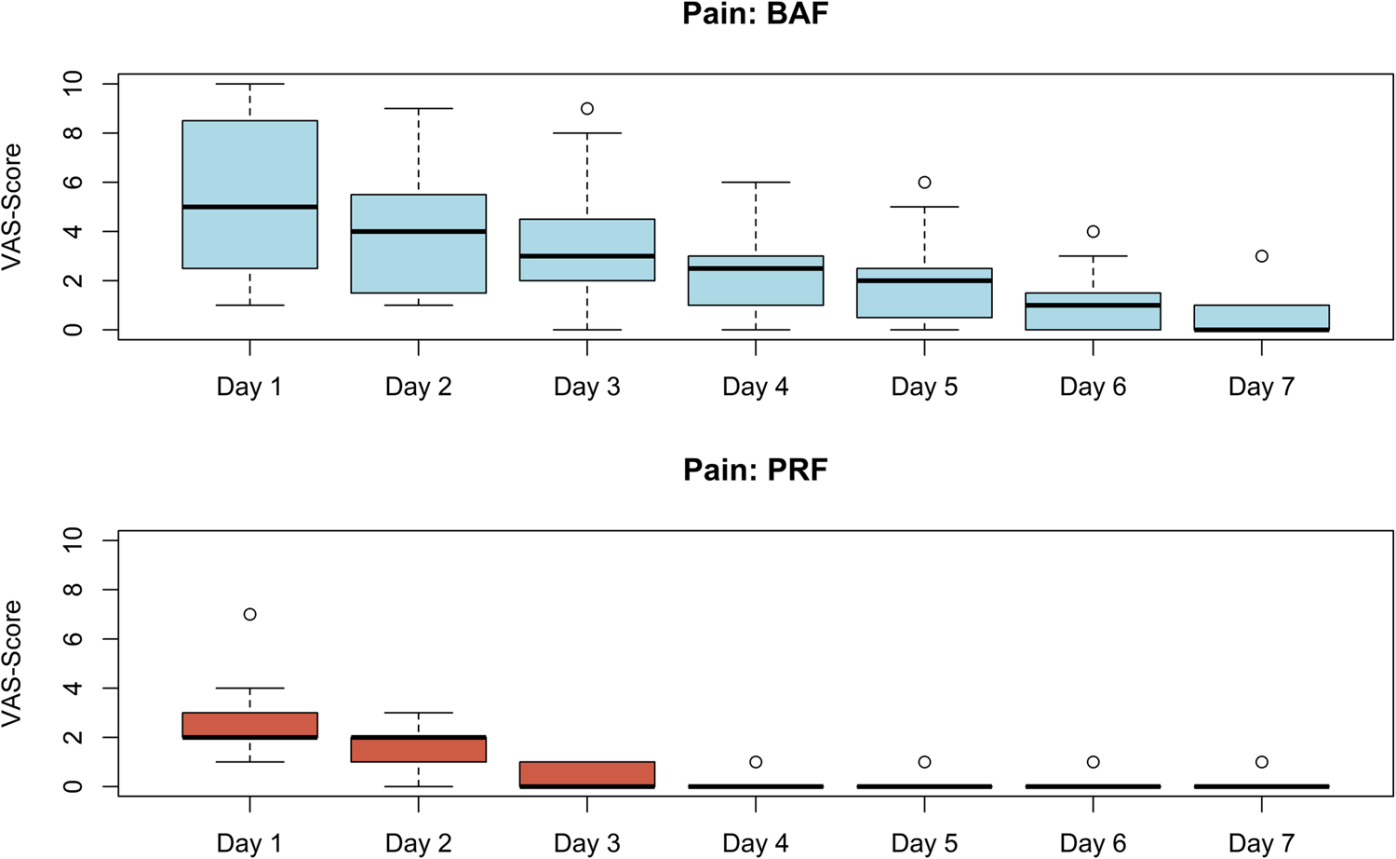
Postoperative pain

**Fig. 8:**
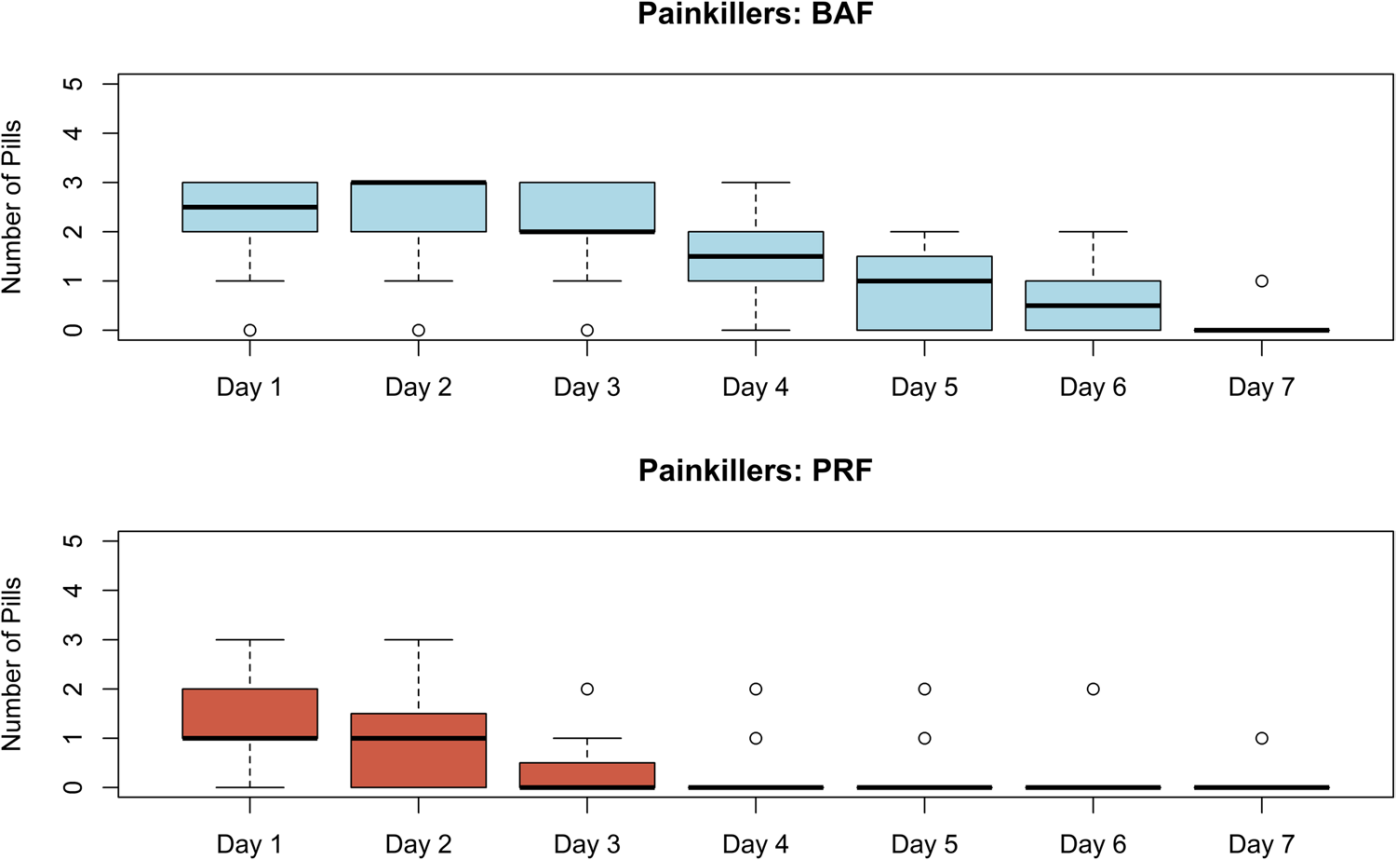
Number of painkillers used per day

Postoperative pain and number of painkillers used per day are presented in Figs. 7 and 8. Pain levels and the number of painkiller medications needed were significantly higher (*p* < 0.001) in the BAF group compared to the group with PRF.

## Discussion

The findings of the present study confirm the initial hypothesis that there are no differences between surgical treatment with a buccal advancement flap and the treatment with platelet-rich fibrin clots/membranes regarding the clinical success rates for the closure of oroantral communication. The final evaluation at the study endpoint on day 21 showed a closure success rate of 90% for both methods demonstrating that the use of platelet rich fibrin (PRF) may be a promising alternative approach for OAC closure.

However, there is inconsistent data about the recommended number and form of PRF clots. The findings of the present study as well as the results of the previous studies by Bilginaylar, Gülsen and Agarwal have indicated high success rates, although a different number of clots was used in each study [15, 23, 24]. In the study by Bilginaylar et al., two platelet-rich fibrin clots were pressed into plugs and placed in the extraction socket for OAC closure [15], whereas Gülsen et al. used six clots for this purpose [23]. Nevertheless, both studies reported a successful healing process. In the study by Agarwal et al. a slightly different protocol is described, where four clots were produced in total, but only three of those were pressed into a cylinder-shaped plug to seal the opening, while the remaining clot was shaped into a membrane which was subsequently folded under the buccal and palatinal mucoperiostal flap for separating the clot from the oral cavity [24]. A similar technique was also used in this present study but instead of three clots only two were used for creating the plug and the other two were used for forming the membrane. However, none of the available studies mentioned above clearly state which number of PRF clots is necessary for achieving successful OAC closure.

Interestingly, the findings of the univariate analysis evaluating the risk factors affecting the defect healing outcome revealed differences between the use of PRF or the buccal advancement flap. For the use of the buccal advancement flap, the patient-related and surgery-related/anatomical risk factors did not differ between successful and failed wound healing outcome. Hence, the failed wound healing observed with a buccal advancement flap may be preferably attributed to patient compliance (nose breathing) and/or surgery-related reasons such as extensive flap tension. In contrast, for the PRF group, the univariate analysis showed significant differences for risk factors such as patient age and defect dimension. In detail, a significant difference between the defect size and success of defect closure was found, suggesting that clinical wound closure may be influenced by the number of platelet-rich fibrin clots used, considering that all patients received the same amount of PRF clots. Consequently, an individualized number of clots depending on the defect size might be the key to an improved outcome for oroantral communication defect closure. This will have to be evaluated in future studies.

Furthermore, the univariate risk factor analysis revealed that patient age seems to be a risk factor for a failed or successful outcome when using PRF. This is consistent with the findings of the studies by Miron et al. (2019) and Mamajiwala et al. which demonstrated that platelet-rich fibrin shows age-related differences regarding the protein content. Specifically, PRF from younger patients exhibits a higher platelet concentration, antimicrobial activity, and a denser fibrin network [25, 26]. Moreover, Yajamanya et al. also found that the fibrous protein in PRF changed with age when density decreased and it became increasingly loose; the number of platelets and white blood cells also decreased [27]. Therefore, structural abnormalities of the PRF in the older age group may have to be compensated when working with this method, for example by using a higher number of clots/plugs, by modifying the centrifugation protocol or by selecting alterative treatment procedures.

The results of the systematic review of Miron et al. evaluating 31 clinical studies highlight the positive effects of PRF on wound healing after regenerative treatment of various soft tissue defects in medicine and dentistry [28]. Platelet-rich fibrin has been described as a natural matrix consisting of various wound healing cell types as well as of proteins and various cytokines, which provide functional and structural support for the regeneration process. Apart from several molecules such as collagen, heparan sulfate, elastin and proteoglycans, some plasma-derived proteins such as fibrin, fibronectin, and thrombospondin are transformed into soft tissue at the surface as well into bone in the residual sockets [29–35].

Although the use of PRF has been reported with excellent clinical outcome, no detailed information is available regarding the wound healing course. The findings of the present study using the wound healing score indices of Landry et al. [20] demonstrate that platelet-rich fibrin and the buccal advancement flap have opposite wound healing courses. Initially, the wound healing was significantly better for the end-to-end anastomosed buccal flap compared to the granulation process of platelet-rich fibrin membrane/plug covering the OAC defects. However, at the endpoint evaluation, the healing effect of the platelet-rich fibrin had caught up with the direct flap adaptation and even showed significantly better final clinical wound healing results. This may be attributed to the fact that wound healing with PRF generally takes longer than the direct adaptation of the wound margins with the buccal flap procedures. The fact that the ultimate score rating was higher with the use of the PRF may possibly be explained by the fact that the fibrin/fibronectin clots and membranes had been transformed into soft tissue that showed similar characteristics as the neighboring areas.

Regarding the evaluation of the secondary outcome measurements, the displacement of the mucogingival borderline also showed a significant difference between the two methods. The displacement of the mucogingival borderline after the buccal advancement flap procedure was to be expected, however, there was also a slight displacement of the mucogingival borderline in the platelet-rich fibrin group. This may be attributed to the remodeling process as a result of bundle bone resorption of the extraction sockets. While the extensive displacement of the mucogingival borderline with the buccal advancement flap occurs due to iatrogenic/surgically induced reasons, the comparatively small shift in the PRF group is based on the physiological remodeling processes.

The findings of this present study also confirm the hypothesis that the presence of pain and the use of painkillers needed was significantly different between the two groups. This is consistent with the findings of Bilginaylar et al. and may either be attributed to the less invasive treatment protocol or to the anti-inflammatory activity and immune regulation effects of the platelet-rich fibrin content [15]. Pain reduction was also reported in separate clinical studies of Choukroun et al. and Kumar et al. in which the platelet-rich fibrin was used as a filling material in extraction sockets [36, 37]. It is also known from other areas of oral surgery that PRF can lead to pain reduction [38–41]. A limitation of this study is that it only provides a comparison between two methods of closure of oroantral communications. As previously mentioned in the introduction, there are other methods such as other local flaps, distant flaps, bone grafts as well as leucocyte and platelet rich fibrin. The widely used and well-established buccal advancement flap was chosen in this study to venture a comparison to PRF, but future studies should consider comparing additional methods as well.

## Conclusions

According to the findings of the current study, the use of platelet-rich fibrin represents a reliable and successful method for closure of oroantral communications. The use of PRF clots for defect filling is associated with low/lowered pain levels, a promising healing pattern and a good clinical soft tissue outcome showing similar tissue characteristics as the adjacent region. However, the defect size and hence the number and size of PRF plugs used is decisive for a successful healing outcome.

## Data Availability

Not applicable.
